# Heterogeneity of Genotype–Phenotype in Congenital Hypofibrinogenemia—A Review of Case Reports Associated with Bleeding and Thrombosis

**DOI:** 10.3390/jcm11041083

**Published:** 2022-02-18

**Authors:** Monika Brunclikova, Tomas Simurda, Jana Zolkova, Miroslava Sterankova, Ingrid Skornova, Miroslava Dobrotova, Zuzana Kolkova, Dusan Loderer, Marian Grendar, Jan Hudecek, Jan Stasko, Peter Kubisz

**Affiliations:** 1 National Centre of Hemostasis and Thrombosis, Department of Hematology and Transfusiology, University Hospital in Martin, Jessenius Faculty of Medicine in Martin, Comenius University in Bratislava, 03601 Martin, Slovakia; simkovamonika@gmail.com (M.B.); jana.zolkova@gmail.com (J.Z.); miroslava.sterankova11@gmail.com (M.S.); inkaskornova@gmail.com (I.S.); miroslava.dobrotova@gmail.com (M.D.); hudecek@unm.sk (J.H.); jan.stasko@uniba.sk (J.S.); peter.kubisz@uniba.sk (P.K.); 2 Biomedical Centre Martin, Jessenius Faculty of Medicine in Martin, Comenius University in Bratislava, 03601 Martin, Slovakia; zuzana.snahnicanova@uniba.sk (Z.K.); dusan.loderer@uniba.sk (D.L.); marian.grendar@uniba.sk (M.G.)

**Keywords:** fibrinogen, hypofibrinogenemia, heterogeneity of phenotype, mutations associated with bleeding and thrombosis

## Abstract

Congenital fibrinogen disorders are diseases associated with a bleeding tendency; however, there are also reports of thrombotic events. Fibrinogen plays a role in the pathogenesis of thrombosis due to altered plasma concentrations or modifications to fibrinogen’s structural properties, which affect clot permeability, resistance to lysis, and its stiffness. Several distinct types of genetic change and pathogenetic mechanism have been described in patients with bleeding and a thrombotic phenotype, including mutations affecting synthesis or processing in three fibrinogen genes. In this paper, we focused on familial hypofibrinogenemia, a rare inherited quantitative fibrinogen disorder characterized by decreased fibrinogen levels with a high phenotypic heterogeneity. To begin, we briefly review the basic information regarding fibrinogen’s structure, its function, and the clinical consequences of low fibrinogen levels. Thereafter, we introduce 15 case reports with various gene mutations derived from the fibrinogen mutation database GFHT (French Study Group on Hemostasis and Thrombosis), which are associated with congenital hypofibrinogenemia with both bleeding and thrombosis. Predicting clinical presentations based on genotype data is difficult. Genotype–phenotype correlations would be of help to better understand the pathologic properties of this rare disease and to provide a valuable tool for the identification of patients who are not only at risk of bleeding, but also at risk of a thrombotic event.

## 1. Fibrinogen’s Structure and Its Function

Fibrinogen is a 340 kDa glycoprotein that circulates in the blood [1]. The fibrinogen molecule is formed from a hexamer that consists of two copies of three nonidentical polypeptide chains (Aα, Bβ, and γ) [2]. Aα chains contain 610 residues, Bβ chains 461 residues, and the major γ chains consist of 411 residues. These polypeptide chains, Aα, Bβ, and γ, are connected by disulfide bridging [3]. Fibrinogen has a characteristic trinodal organization, consisting of a single central and two distal nodes [4]. The molecule has a 45 nm elongated structure with two outer D domains, which are connected to a central E domain by a coiled-coil segment [5]. Three independent genes encoding fibrinogen are clustered on chromosome 4 (region 4q31.3) [6]. Each chain of fibrinogen is encoded by a separate gene. The gene coding for the fibrinogen Aα chain (*FGA*) has a 7.6 kb size and consists of 6 exons, the Bβ chain gene (*FGB*) has 8 exons and occupies an 8.0 kb sized region, and the γ chain gene (*FGG*) encompasses an 8.5 kb region and consists of 10 exons [7]. Normal fibrinogen blood levels vary and are typically given as 1.8–4.2 g/L [8]. Fibrinogens belong to the acute-phase proteins whose levels increase in response to different stressful situations, such as tissue injury, inflammation, and the accompanying release of cytokines. Upregulation of fibrinogen expression is controlled by interleukin 6 (IL 6) and the glucocorticoid signaling pathways [9]. This causes a rapid increase in fibrinogen plasma levels after clotting events or bleeding, and in order to support wound healing [10]. Contrarily, transforming growth factor β (TGF–β), and cytokines IL4, IL10, and IL13 are negative regulators of transcription [9]. Fibrinogen is dominantly expressed in hepatocytes. However, extrahepatic production has been demonstrated in epithelial cells from the lungs, intestine, and cervix. For some years, biosynthesis of fibrinogen by megakaryocytes has been discussed, but it is widely believed that the fibrinogen present in alpha granules of thrombocytes originates primarily from plasma uptake [11].

## 2. Congenital Hypofibrinogenemia—Characterization, Classification, and Clinical Phenotype

**Figure 1:**
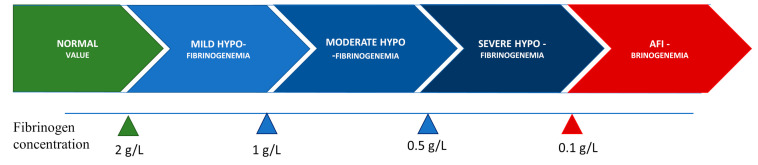
Classification of hypofibrinogenemia according to fibrinogen concentration.

Congenital hypofibrinogenemia is a quantitative fibrinogen disorder characterized by a proportional decrease in functional and antigenic fibrinogen levels [12,13]. On the basis of the level of fibrinogen concentration, hypofibrinogenemia is classified as severe, moderate, or mild. Certain hypofibrinogenemic variants result in liver disease, i.e., fibrinogen storage disease (FSD), which is caused by the intrahepatic accumulation of fibrin [14]. The classifications according to fibrinogen concentration levels are listed in Figure 1.

Hypofibrinogenemia has diverse phenotypical variability, which can range from no manifestations to bleeding and/or thrombosis. Hypofibrinogenemic patients have a lower risk of bleeding than patients with afibrinogenemia [15]. Patients with hypofibrinogenemia are frequently asymptomatic and are diagnosed during routine laboratory testing, before invasive procedures, or in the setting of familial explorations. Hypofibrinogenemia can lead to a milder bleeding pattern, with surgery and trauma, with related bleeding significantly more common than spontaneous events (80% vs. 20%). The most typical bleeding symptoms are menorrhagia, hemorrhage from mucosal tracts, hemarthroses, hematomas, and GI bleeding [11]. Abnormalities of the fibrinogen molecule have been implicated in various adverse pregnancy outcomes, like placental abruption, postpartum hemorrhage, and spontaneous abortion, highlighting the role of fibrinogen in implantation and placentation [16]. Hypofibrinogenemic patients when exposed to other thrombotic risks factors, biological or acquired, they can also develop thrombosis in venous and/or arterial sites. Thus, clinical manifestations are not only the direct result of the fibrinogen level in circulation but also the result of additional personal, genetic, and environmental factors, such as smoking, use of oral contraceptives, immobilization, comorbid conditions, hormonal status, further thrombophilia and bleeding risk factors, amongst others.

## 3. Fibrinogen’s Role in the Pathophysiology of Thrombosis and Bleeding in Hypofibrinogenemia

Fibrinogen plays a crucial function in controlling bleeding after vascular injury by providing the support for platelet aggregation and the substrate for fibrin clotting [17]. Via the activated form of glycoprotein IIb/IIIa (known as integrin αIIbβ3), fibrinogen creates a scaffold for the aggregation of platelets. As a part of the rapid primary hemostatic response, thrombocyte aggregation via fibrinogen crosslinking contributes to the creation of an initial hemostatic barrier after blood vessel injury [18]. Afterwards thrombin is activated on surface of platelets and fibrinogen is converted into insoluble fibrin [19]. The last step in the blood clotting cascade necessary for hemostasis and thrombosis is the conversion of fibrinogen to fibrin [20]. Fibrinogen’s conversion to fibrin stops bleeding by providing the insoluble matrix of the blood clot [21]. As the fibrin is formed, the serine protease thrombin rapidly cleaves fibrinogen, releasing two fibrinopeptides, i.e., fibrinopeptide A (FpA, residues 1–16) and fibrinopeptide B (FpB, residues 1–14), from the N-terminal of the Aα and Bβ chains, respectively, and converts fibrinogen into fibrin monomers [22]. Fibrin monomers spontaneously polymerase, resulting in the formation of thicker fibers and an insoluble multistranded and branched fiber network that entangles platelets to form a blood clot, blocking the damaged blood vessel and preventing further bleeding [23,24].

**Figure 2:**
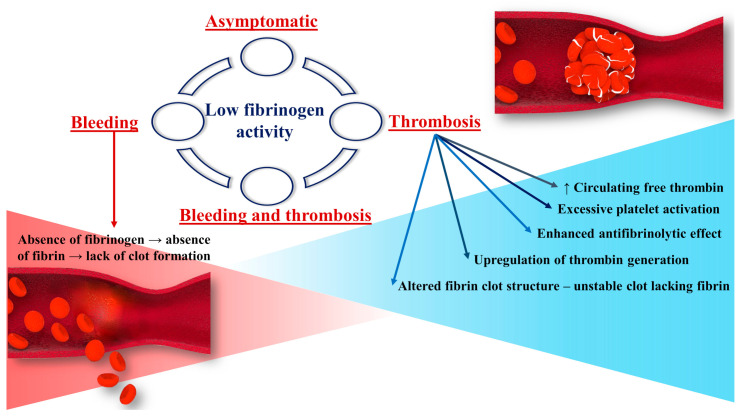
Pathogenesis of thrombosis and bleeding in association with low fibrinogen activity.

Thus, the absence of normal fibrinogen leads to the disruption of said mechanisms and thus to bleeding complications (Figure 2). According to the literature data, the severity and pattern of clinical manifestations of bleeding are dependent on the fibrinogen levels [25]. With fibrinogen levels of around 1.0 g/L, patients with hypofibrinogenemia are usually asymptomatic. In theory, these levels are high enough to protect against bleeding and maintain pregnancy [26]. The bleeding phenotype usually develops in patients with fibrinogen levels lower than 1.0 g/L. A mean activity level of fibrinogen of at least 0.7 g/L prevents spontaneous hemorrhage [27].

Fibrinogen mediates the modulation of coagulation and fibrinolysis through thrombin binding, conferring antithrombin activity, and through FXIII, plasminogen, antiplasmin, and tissue-type plasminogen activator (t-PA) [28]. Fibrinogen contributes to various pathological events including thrombosis due to decrease of plasma concentration of fibrinogen, its changed structural properties, or from the effect of polymorphisms on clot stiffness, permeability, and resistance to lysis (Figure 2) [10]. The reasons for the increased thrombotic risk in congenital fibrinogen disorders (CFD) are not entirely understood [29]. Thrombosis can develop in both arterial and venous sites. These events are less common in hypofibrinogenemic patients than in afibrinogenemic patients. The low level of circulating fibrinogen in hypofibrinogenemia is generally thought to be enough to lower the risk of developing thrombosis, which is more frequently described in afibrinogenemia. It is important to mention that low fibrinogen levels do not compensate for a hypercoagulable state [27].

One explanation for the increased risk of a thrombotic event in hypofibrinogenic patients is that circulating thrombin levels increase in association with fibrinogen deficiency. Thrombin is normally sequestered by the developing fibrin, thus reducing the amount of free thrombin in circulation. Free thrombin has the potential effect of platelet activation [29]. Fibrin deficiency reduces its antithrombin effect, causing an increase in circulating thrombin levels. Free thrombin, which is not trapped in the fibrin clot, activates platelets and stimulates the migration and proliferation of smooth muscle cells, leading to large and loose platelet thrombi [27]. Fibrin can also lower thrombin generation. Therefore, an absence or reduction of functional fibrinogen can be responsible for increased thrombin activity [29]. Soluble fibrinogen also competitively reduces platelet adhesion to immobilized fibrinogen. However, hemostasis in patients with quantitative fibrinogen disorders enables adequate thrombus formation. As a result of the lack of fibrin in clots, they become unstable and have tendency to embolize [30].

The clinical data are controversial as regards fibrinogen replacement therapy. It has been suggested that treatment with fibrinogen concentrates may be a risk factor for thrombosis. However, it should be noted that there is no clear evidence of a direct link between fibrinogen concentrate administration and the development of thrombosis. The pathogenesis at the basis of the paradoxical thrombotic tendency in patients with fibrinogen deficiency is likely multifactorial, depending on different exogeneous and endogenous risk factors, such as genetic thrombophilia, immobilization, pregnancy, surgery, or trauma. Thrombosis is described in more than one-third of hypofibrinogenemic patients after surgery, delivery, puerperium, and trauma. Thrombophilic mutations (i.e., protthombin G20210 and factor V Leiden) [19] have been reported in a small number of patients with CFD.

## 4. Review of Mutations Associated with Hypofibrinogenemia and Both Bleeding and Thrombotic Phenotype

Quantitative fibrinogen deficiencies are characterized by the concomitant reduction or absence of coagulant activity and immunoreactive proteins. Complete mutational screening of all three fibrinogen genes (*FGA*, *FGB*, *FGG*) is required for the molecular diagnosis of congenital fibrinogen disorders [31]. While many of the polymorphisms and minor alterations do not influence fibrinogen’s structure, function [32], or the multimeric form of fibrin, and are thus seen to be neutral, some of the more significant changes have been shown to influence fibrinogen’s function, structure, or both [33]. The spectrum of abnormal changes in molecular structure is broad, resulting into several subtypes of fibrinogen disorders with specific clinical and biological features [14].

We distinguish two main classes of causative mutations: mutations producing abnormal protein chains, which are retained inside the cell; and null mutations with no protein production at all. Hypofibrinogenemia is generally caused by heterozygosity for these mutations.

We searched for human fibrinogen variants associated with hypofibrinogenemia and both bleeding and thrombosis in the mutation database GFHT (French Study Group on Hemostasis and Thrombosis). In this database, new variants in all three genes, i.e., *FGA*, *FGB,* and *FGG,* for fibrinogen are regularly added. The GFHT database is available via the Study Group on Hemostasis and Thrombosis: http://www.geht.org/databaseang/fibrinogen, accessed on 6 October 2021, which lists all fibrinogen variants identified to date in patients with afi-, hypo-, dys-, and hypodysfibrinogenemia. As of 6 October 2021, the GFHT database lists 1215 molecular abnormalities associated with fibrinogen.

We looked at all of the gene mutations in all three fibrinogen genes. Overall, in this database, we found 69 such mutations. Among them, 24 mutations resulted in afibrinogenemia, 15 in hypofibrinogenemia, 22 in dysfibrinogenemia, with the least common disorder being hypodysfibrinogenemia, which is caused by eight mutations. Most mutations were detected in the *FGA* gene. The lowest number of variants was found in the *FGB* gene. In Table 1, all genetic variants in *FGA*, *FGB*, and *FGG* associated with both bleeding and thrombosis are summarized according to different CFD types.

On the basis of the GFHT database, a total of 15 mutations responsible for both a hypofibrinogenemia and a thrombotic and bleeding phenotypes were identified: three in *FGA*, five in *FGB,* and seven in *FGG*. Eight mutations were heterozygous, two homozygous, and five cases were the result of compound heterozygosity. Thirteen were localized in exons and two in introns. Most mutations were caused by genetic changes in exon 8 of *FGG*. The majority of patients with hypofibrinogenemia and mutations determining both the bleeding and thrombotic phenotype experienced venous thrombosis. The most common were pulmonary embolism, deep venous thrombosis, cerebral venous thrombosis, etc. Thrombotic events in the arterial circulation, such as myocardial infarction, ischemic stroke, peripheral arterial thrombosis, or arterial thrombosis at other sites, were less frequent. Thrombotic recurrences were mainly present in the venous territory. In addition, certain mutations were reported to be associated with at least with one miscarriage.

**Figure 3:**
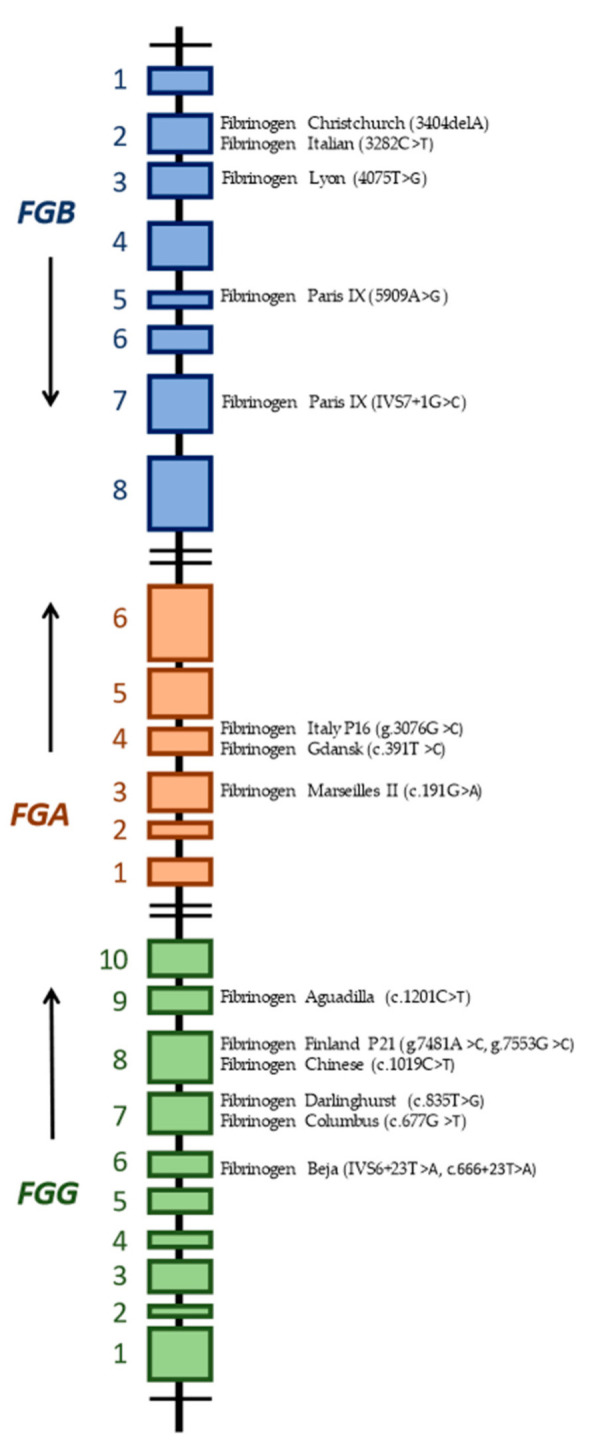
Schematic diagram of *FGA*, *FGB,* and *FGG* genes and the location of individual mutations associated with hypofibrinogenemia and thrombotic and bleeding events.

Table 2 lists the mutations leading to hypofibrinogenemia associated with both bleeding and thrombotic events and summarizes the available clinical data. We present the different bleeding and thrombotic events for each mutation and the associated miscarriages. We also provide the number of patients studied, the asymptomatic patients with the described mutations, and the number of patients with thrombotic and hemorrhagic events. Figure 3 is a schematic diagram of the mutations in congenital hypofibrinogenemia and their locations in fibrinogen genes.

## 5. Mutations in the FGA Gene

### 5.1. Fibrinogen MARSEILLES II

A 27-year-old Portuguese woman with a history of spontaneous bleeding throughout childhood and with severe central venous thrombosis in adulthood was studied by Soares et al. On both occasions, the woman underwent a thorough laboratory assessment and was diagnosed with hypofibrinogenemia (<0.8 g/L) and with protein S deficiency.

After spontaneous gastrointestinal bleeds as a child, she was laboratory tested for bleeding disorders, which revealed a fibrinogen deficiency. The patient was hospitalized because of acute atraumatic pain and swelling on the right calf at the age of 27. A recent occlusive thrombosis involving external iliac, common femoral, superficial femoral, and right popliteal veins was revealed with venous ultrasound with Doppler imaging. After six months of treatment with low molecular heparin (enoxaparin), the thrombophilia work-up was repeated, confirming the persistence of hypofibrinogenemia, with a normal quantitative protein S value (74%) and a lower level of activity (32%). Genetic testing finally revealed, revealing a pathogenic heterozygous mutation in the *FGA* gene c.191G>A, p.Cys64Tyr. This mutation is known as Fibrinogen Marseilles II and is a pathogenic variant of hypofibrinogenemia that leads to alterations in fibrinogen conformation and secretion.

In conclusion, the presented case was characterized by spontaneous bleeding during childhood caused by inherited hypofibrinogenemia due to heterozygous mutation in the *FGA* gene. She later suffered from deep vein thrombosis due to deficient proteins S activity and the use of hormonal therapy with the objective of menstrual flow control due to metrorrhagia secondary to hypofibrinogenemia and family planning. This highlights the need for proper drug management in similarly affected patients and the risk of both bleeding and thrombotic complications [34].

### 5.2. Fibrinogen ITALY P16

A 52-year-old Italian man with moderate hypofibrinogenemia had a history of multiple events of deep vein thrombosis and colon bleeding, as reported by Asselta et al. Heterozygous missense mutation was identified using genetic analysis in exon 4 of the *FGA* gene g.3076G>C (p.Arg129Pro native protein variation, p.Arg110Pro mature protein variation). No secretion defect of the hexametric fibrinogen molecule was related to this mutation. The p.Arg129Pro fibrinogen seemed to imitate the wild-type one perfectly [17].

### 5.3. Fibrinogen GDANSK

Cases of a missense mutation in *FGA* exon 4 c.391c.T>C (p.Ser131Pro in α chain without the signal peptide) associated with hypofibrinogenemia were reported in a Polish family by Mital et al. A 29-year-old male with lifelong bleeding tendency (trauma-induced bleeding, bleeding after dental care) developed enormous penile hematoma after penis correction surgery. His clottable fibrinogen measured using the Clauss method was 0.4 g/L. His son, sister, and father were also diagnosed with hypofibrinogenemia. The patient’s sister, a 24-year-old woman, had a history of hemorrhage including prolonged bleeding after skin cuts or injury, easy bruising, and heavy bleeding during surgical removal of an ovarian cyst. While on hormonal contraceptives for four months and after a long trip, she developed occlusions of the left calf and popliteal veins. Her functional fibrinogen levels were recorded from 0.35 to 0.53 g/L. Thrombophilia screening was negative [42].

## 6. Mutations in the FGB Gene

### 6.1. Fibrinogen CHRISTCHURCH V

A 20-year-old female from New Zealand was sent for a diagnostic test at 12 years of age due to recent bruising over her legs. She suffered a sudden episode of seizure activity concomitant with a thrombotic stroke that was diagnosed based on multiple imaging studies from the age of 14 months. A hematological examination performed at the time revealed a low antigenic and functional fibrinogen and no other thrombophilic state. This case was reported by Brennan et al. Her brother and father also had hypofibrinogenemia, and they carried the same genetic defect that was present in proposita. However, they had been asymptomatic up until then. The proposita was heterozygous for a novel mutation—a single nucleotide deletion at codon 58 of the Bβ chain (3404delA, p. Lys88FrameshiftStop). This mutation is capable of this as the deletion and consequent frame shift occurs at codon Bβ58 and results in a unique nonfibrinogen sequence and a premature chain termination 41 residues later. This prevents the formation of disulfide rings, which are crucial for the formation of the coils that link the central E region to the peripheral D regions. From this translation product, no viable molecules can be assembled. Whilst it is clear that Bβ gene codon 58 frame shifts lead to the hypofibrinogenemia, the cause and pathogenesis of the ischemic stroke in childhood is less obvious. Other than hypofibrinogenemia, the proposita did not have any known thrombotic risk factors, and it therefore seems probable that this hypofibrinogenemic mutation contributed to the thrombotic event [43].

### 6.2. Fibrinogen ITALIAN

A 26-year-old Milan woman with a history of permanent bleeding symptoms was diagnosed with severe hypofibrinogenemia after prolonged oozing after a tooth extraction, as reported by Asselta et al. She had suffered six spontaneous abortions. At the age of 60, she developed thrombotic occlusion of the anterior right tibial artery at the third distal end of the dorsal metatarsal artery, with the dorsalis pedis artery being barely evident. The anterior tibial artery of the left extremity was thrombosed. No other thrombophilic state, except an FV Leiden mutation, was present. The genetic basis of severe hypofibrinogenemia was analyzed and the patient turned out to be a compound heterozygote for 2-point mutations in the *FGB* gene [44]. One mutation was a novel T>A transversion in exon 4 at position 5157, which was thought to cause the Leu172Gln substitution. The next mutation was in exon 2, i.e., a C>T transition at position 3282, leading to a nonsense mutation at residue 17 in the fibrinogen Bβ chain gene. The mutation Arg17Stop had been previously documented in the homozygous state in an Iranian patient with afibrinogenemia. Assembly and secretion in Fibrinogen Italian (the mutant Bβ-Leu172Gln fibrinogen) were not disrupted. Inspection of the nucleotide sequence surrounding the mutation suggested a possible role of pre-messenger RNA (mRNA) splicing, resulting in a truncated Bβ chain, lacking approximately 70% of the C-terminal region [4,35].

### 6.3. Fibrinogen LYON

A 52-year-old patient reported three abortions, bleeding after dental extraction, and two deliveries in her history. Hypofibrinogenemia was diagnosed after postpartum bleeding and was named fibrinogen Lyon. This case was reported by Hanss et al. The proband’s three daughters were also diagnosed with hypofibrinogenemia between the ages of 3–5 by systematic evaluation. Easy bruising, epistaxis, and two miscarriages were reported by the oldest daughter who also developed cerebral venous occlusion while using oral contraception for two years. The second daughter reported easy bruising. For the proposita and her younger daughter, a heterozygous 4075T>G transversion was detected in the Bβ chain gene, predicting the p.Met148Lys missense mutation. Informed consent and/or DNA were not available for the two other daughters. The p.Met148Lys mutation was found in the initial part of the triple helix. The integrity and structure of the C-terminal βD domain should not be influenced. For the correct assembly of the fibrinogen chains, the proper assembly of the coiled-coil region is required. The substitution of a nonpolar residue by a positively charged one at position 118, near the coiled-coil domain, can destabilize the coiled-coil assembly [36].

### 6.4. Fibrinogen PARIS IX

Mutations named Fibrinogen Paris IX were reported in a 36-year-old woman with hemorrhagic, thrombotic, and pregnancy complications and moderate hypofibrinogenemia (fibrinogen function 0.7 g/L) by Horellou et al. She reported recurrent nose bleeding during infancy, a lifelong history of menorrhagia, and easy bruising. Ten days after the delivery of a dead fetus by emergency Caesarean section, she developed distal vein thrombosis. Genetic tests found both a heterozygous missense mutation in exon 5 of the *FBG* gene c. 509A>G (p.Tyr266Cys) and a heterozygous IVS7 + 1G>C transversion. This mutation possibly affects exon 8 splicing. The p.Tyr266Cys mutation occurs in the second β sheet and can lead to destabilization of the structure. There is the possibility that incorrect disulfide bonding occurs in the chain that is mutated. In association with hypofibrinogenemia, the p.Tyr266Cys mutation strengthens the conclusion that the proper organization of the β sheet structure of the Bβ chain portion is needed for fibrinogen assembly and/or secretion [37].

## 7. Mutations in the FGG Gene

### 7.1. Fibrinogen BEJA

A 50-year-old man from a blood-related marriage was diagnosed with severe hypofibrinogenemia at the age of 40 after an examination due to atypical bleeding after dental care. Two years later, he developed acute myocardial infarction. The patient was found homozygous for a novel intronic mutation in intron 6 of the *FGG* gene IVS6 + 23T>A (c.666 + 23T>A). The mutation was designated Beja and reported by Amri et al. The substitution created a higher donor splice site score, resulting in the insertion of 19 base pairs into the mutated mRNA. The reduction in fibrinogen level could be explained by either the accumulation or the proteolysis of the truncated λ chain in the endoplasmic reticulum or degradation of mutated mRNA by the nonsense-mediated mRNA decay mechanism. In vitro testing indicated that the mutation that leads to fibrinogen Beja affects the fibrinogen secretion process [38].

### 7.2. Fibrinogen DARLINGHURST

Fibrinogen Darlinghurst is a missense mutation c.835T>G (p.Trp279Gly) in the fibrinogen gamma chain. It was first described in a patient with both thrombotic and bleeding complications associated with severe hypofibrinogenemia. The patient was a Turkish female with signs of right heart failure and was found to have pulmonary hypertension secondary to thromboembolic disease. She experienced multiple abortions and bleeding after dental care. The patient showed no laboratory evidence of known inherited or acquired thrombophilia. DNA sequencing showed that the patient was homozygous for a single nucleotide substitution in exon 7 of the fibrinogen γ gene (*FGG* c.835T>G) leading to a tryptophan to glycine change at residue 253 of the mature γ chain (residue 279 from the initial codon). Researchers performed tPA/glu-plasminogen catalyzed fibrinolysis and found that fibrin clots from the patient lysed at roughly half the rate of the controls. This led to the conclusion that the mutant protein was resistant to lysis. This mutation, in heterozygous form, was also found in her son, who showed a moderately reduced fibrinogen level and, at the time of examination, was asymptomatic [1].

### 7.3. Fibrinogen COLUMBUS

A novel mutation causing hypofibrinogenemia in a family associated with thrombotic event was documented by Davis et al. The proband was a two-year-old boy. He and his twin were both found to be hypofibrinogenemic with a Clauss’ fibrinogen of 0.3 g/L at birth. Postpartum, his twin was diagnosed with subdural and subarachnoid hemorrhaging and diffuse areas of hypoxic ischemia. The twin’s neurological function continually deteriorated, resulting in death at seven months of age due to intracranial thrombotic and hemorrhagic events. Their mother had a history of deep vein thrombosis. She displayed borderline hypofibrinogenemia. DNA sequencing revealed a heterozygous GGC → GTC mutation in exon 7 (c.G677T) of the fibrinogen γ gene. This γ200Gly → Val mutation was found in both mother and the living son and was described as Fibrinogen Columbus. Expression of the variant chain in plasma fibrinogen was not detected. The γ200Gly → Val mutation appears to be the direct cause of hypofibrinogenemia. It segregates with low functional and physical fibrinogen levels in affected individuals. Both twins and their mother were heterozygous for the factor V Leiden mutation. The mother also displayed a MTHFR C677T polymorphic mutation. The homocysteine level was not provided. In conclusion, the γ200Gly → Val mutation is the cause of hypofibrinogenemia and may contribute to the cumulative effect of other (factor V Leiden, MTHFR C677T, and *FGG*—H2) thrombophilic mutations in their kindred [39].

### 7.4. Fibrinogen FINLAND P21

In some cases, the original paper did not fully describe the clinical features of the patient. This is also the case for a patient with fibrinogen Finland P21 for which only partial data were available.

Hypofibrinogenemia, named fibrinogen Finland 21, was diagnosed in a woman during examinations after suffering an episode of thrombosis. The location of thrombosis and the presence of another thrombophilic state were not reported. This woman did not have any history of bleeding; however, her three children had minimal unprovoked bleeding. Therefore, we present the case in our review. The researchers documented a complete lack of fibrinogen while carrying the p.ThR303Pro *FGG* mutation (in combination with p.Asp327His in *FGG* or alone), suggesting total secretion impairment. p. Asp327His fibrinogen was adequately released into the medium, thus showing that hypofibrinogenemia in this patient can be explained by the sole p.ThR303Pro mutation, whereas p. Asp327His is probably a neutral variant [17].

### 7.5. Fibrinogen CHINESE (JIUJIANG)

Nia et al., published a case report of a 44-year-old woman with moderate hypofibrinogenemia and previous abnormal childbearing history experienced vaginal bleeding for two months. She was given norethindrone and curettage was performed. After the procedure, the patient suffered from a severe headache and vomiting. MRI imaging revealed multiple cortical infarctions and cortical venous thrombosis. The *FGG* heterozygous mutation c.1019C>T (p.Thr340Ile) in exon 8 was detected by Sanger and whole exome sequencing. The patient’s two children and her sisters had the same mutation. The children had mild hypofibrinogenemia and the sister exhibited normal fibrinogen levels. It was determined as autosomal dominant inheritance, cosegregation of the mutation, and disease in the family. This mutation leads to severe impairment of the fibrin monomers polymerization, leading to low clottability and the formation of many soluble fibrins, resulting in a significantly higher risk of thrombosis [40].

### 7.6. Fibrinogen AGUADILLA

The molecular peculiarity of FSD associated with hypofibrinogenemia and hepatic inclusions caused by the impaired release of abnormal fibrinogen, which accumulated and aggregated in the hepatocellular endoplasmic reticulum, was examined by Casini at al. in family with fibrinogen Aguadilla from Switzerland. The proband, a four-year-old, otherwise healthy, girl, with no history of marked bleeding episodes or thrombosis, was found to be heterozygous for fibrinogen Aguadilla: p.Arg401Trp in exon 9 of *FGG* (c.1201C>T). Her mother reported heavy menstrual bleeding and severe hemorrhage after Caesarean section. She was diagnosed with intracranial sinus venous thrombosis despite the thrombophilia tests being negative. DNA was not available from the mother for a further fibrinogen mutation analysis. An additional finding in the mother’s genetic tests was a heterozygosity for HFE p.C282Y—hereditary haemochromatosis. Fibrinogen Aguadilla is assembled, accumulates, and aggregates in the endoplasmic reticulum. Only the normal γ chains were detected in patient’s circulation. The p.Arg401Trp mutation replaces the large, positively charged arginine residue of the wild-type protein with the hydrophobic, ring-structured tryptophan residues. This substitution results in the loss of five hydrogen bonds and the gain of one hydrogen bond in the vicinity of the mutation. Thus, fibrinogen Aguadilla leads to alterations in fibrinogen assembly. However, as no abnormal fibrinogen can reach the circulation in the setting of FSD, the role of the mutation in the development of thrombosis is unclear [41].

## 8. Discussion

Despite the large amount of information regarding the epidemiology and genetics of inherited fibrinogen disorders, which allow us to better understand the abnormalities in fibrinogen’s molecular structure and more precisely define the clinical presentations of these disorders, predictions concerning individual phenotypes remain difficult. Congenital fibrinogen abnormalities have a huge diversity in terms of expressivity and penetrance. The clinical phenotype of patients with hypofibrinogenemia is highly heterogeneous. In hypofibrinogenemia, certain patients have significant bleeding episodes; in others, minor bleeding may occur, whilst some remain asymptomatic their whole life. Paradoxically, however, for disorders with a tendency to hemorrhage, thrombotic events are reported. Some patients with hypofibrinogenemia develop venous or arterial thrombosis in the presence or absence of fibrinogen replacement therapy. Thrombosis and obstetrical complications occur in hypofibrinogenemic patients, and their management requires a multidisciplinary approach.

Data on quantitative fibrinogen disorders and both the bleeding and thrombotic phenotypes are very limited and only available from a small number of patients in the form of case reports. The literature demonstrates a good correlation between fibrinogen level and bleeding phenotype in hypofibrinogenemia. However, this was not entirely true in the documented case reports. For example, fibrinogen Beja, in a proband with severe fibrinogen deficiency, did not cause spontaneous bleeding. Despite the serious deficiency, the patient only suffered from provoked bleeding during dental treatment. On the other hand, the young woman with documented fibrinogen Marseilles II with a fibrinogen level of 0.8 g/L exhibited spontaneous gastrointestinal bleeding. However, no bleeding events were documented in this case during LMWH treatment for deep vein thrombosis. It is possible that the synergistic effect of other factors, such as the presence of a thrombophilic state, e.g., a protein S deficiency, may have had some protective effect before bleeding events in this patient. This case demonstrates that research for other risk factors should be performed when thrombophilia occurs in a patient with hypofibrinogenemia and illustrates the importance of global coagulation testing and the need for the comprehensive approach in these patients. The severity and location of the bleeding were remarkably diverse in the aforementioned case reports. The location of the thrombotic event was also different. This variability in phenotype makes clinical and genetic counselling more difficult. On the basis of these data, it is exceedingly difficult to predict how the disease will manifest itself with weak segregation, even among family members with the same mutations. It is also vital to note that even an individual who is asymptomatic at the time of diagnosis and carries a mentioned causal mutation may be at risk of adverse consequences during their lifetime.

A more accurate determination of changes in the molecular structure, properties, and amount of fibrinogen and their relationship with the clinical phenotype will help physicians better understand the pathophysiology of the defect and help predict the clinical manifestation of a given molecular deviation in the future. Because CFDs are rare diseases, there is insufficient information in the scientific literature to explore exactly how the disease will manifest. Whether CFD exhibits a bleeding or thrombotic phenotype depends on different exogeneous and endogenous risk factors. We hope that this review will increase research interest in this topic so that, in the future, we will better understand how bleeding and thrombotic complications in patients with hypofibrinogenemia develop. To improve patient identification and assess the risk of adverse outcomes, further studies on larger groups of patients are required.

## Figures and Tables

**Table 1:** Number of mutations in *FGA*, *FGB*, and *FGG* genes associated with both the bleeding and thrombotic phenotype summarized according to different CFD types.

Type of CFD	Variants in *FGA*	Variants in *FGB*	Variants in *FGG*	Total Number of Variants
Afibrinogenemia	14	6	4	24
Hypofibrinogenemia	3	5	7	15
Dysfibrinogenemia	12	2	8	22
Hypodysfibrinogenemia	5	0	3	8
Total number	34	13	22	69

CFD—congenital fibrinogen disorder; *FGA*—the gene coding for the fibrinogen α chain; *FGB*—the gene coding for the fibrinogen β chain; *FGG*—the gene coding for the fibrinogen γ chain.

**Table 2:** Description of clinical studies and fibrinogen mutations in congenital hypofibrinogenemia associated with bleeding and thrombotic manifestations [2,22,34,35,36,37,38,39,40,41].

Name/Origin	Mature Protein Variation	Native Protein Variation	cDNA	Gene Status	e–i	Numbers of Studied Patients/Numbers of Patients with Thrombosis	Bleeding Complications	Numbers of Thrombotic Complications and Obstetrical Problems	Localization of Thrombosis	Other Thrombophilic States
Fibrinogen Aα Chain Mutations Associated with Hypofibrinogenemia and Bleeding and Thrombosis										
MARSEILLES II	Aα(45)Cys˃Tyr	p.Cys64Tyr	c.191G˃A	Heterozyg.	e–3	2/1	Spontaneous digestive tract bleeding Metrorrhagia	1x External iliac VT, common femoral, popliteal	External iliac VT, common femoral, popliteal	Protein S deficiency
ITALY P16	Aα(110)Arg˃Pro	p.Arg129Pro	g.3076G˃C	Heterozyg.	e–4	1	Colon bleeding	Repetitive DVT	DVT	No other thrombophilic state
GDANSK	Aα(112)Ser˃Pro	p.Ser131Pro	c.391T˃C	Heterozyg.	e–4	4/1	Easy bruising, bleeding after surgery	1x Left popliteal and calf veins	Left popliteal and calf veins	No other thrombophilic state
Fibrinogen Bβ Chain Mutations Associated with Hypofibrinogenemia and Bleeding and Thrombosis										
CHRISTCHURCH V	Bβ(58)Lys˃frameshift–stop	p.Lys88Frameshift Stop	3404delA	Heterozyg.	e–2	3/1	Easy bruising	1x IS	IS	No other thrombophilic state
ITALIAN	Bβ(172)Leu˃Gly	p.Leu202Gly	3282C˃T	Compound	e–2	1	Bleeding after dental care	6x Miscar., 1x tibial artery thrombosis	Tibial artery thrombosis	Heterozygous Factor V Leiden
LYON	Bβ(118)Met˃Lys	p.Met148Lys	4075T˃G	Heterozyg.	e–3	2/1	Easy bruising, bleeding after dental care, post—partum bleeding	3x Miscar.	Miscar.	x
PARIS IX	Bβ(236)Tyr˃Cys	p.Tyr266Cys	5909A˃G IVS7+1G˃C	Compound Compound	e–5 i–7	1	Easy bruising, epistaxis, menorrhagia	1x Miscar., 1x DiVT	Miscar, DiVT	x
Fibrinogen γ Chain Mutations Associated with Hypofibrinogenemia and Bleeding and Thrombosis										
BEJA			IVS6+23T˃A (c.666+23T˃A)	Homozyg.	i–6	1	Bleeding after dental care	1x MI	MI	No other thrombophilic state
DARLINGHURST	γ(253)Trp˃Gly	p.Trp279Gly	c.835T˃G	Homozyg.	e–7	2/1	Bleeding after dental care	CHTED, repetitive miscar.	CHTED, miscra.	No other thrombophilic state
COLUMBUS	γ(200)Gly˃Val	p.Gly226Val	c.677G˃T	Heterozyg.	e–7	2/2	x	1x DVT	DVT	Heterozygous Factor V Leiden, MTHFR C677T mutations
FINLAND P21	γ(277)Thr˃Pro γ(301)Asp˃His	p.Thr303Pro p.Asp327His	g.7481A˃C g.7553G˃C	Compound Compound	e–8 e–8	4	x	x	x	x
CHINESE	γ(314)Thr˃IIe	p.Thr340Ile	c.1019C˃T	Heterozyg.	e–8	5/1	Metrorrhagia	1x CVT	CVT	x
AGUADILLA	γ(375)Arg˃Trp	p.Arg401Trp	c.1201C˃T	Heterozyg.	e–9	1/1	x	x	x	x

FVT—femoral vein thrombosis; PE—pulmonary embolism; Miscar.—miscarriages; LI—leg ischemia; IS—ischemic stroke; DVT—deep vein thrombosis; DiVT—distal vein thrombosis; VT—vein thrombosis; CVT—cerebral venous thrombosis; MI—myocardial infarction; CHTED—chronic thromboembolic disease.
